# Factors Associated With Delay in Seeking Medical Care Among Breast Cancer Patients in Bangladesh

**DOI:** 10.7759/cureus.113946

**Published:** 2026-08-04

**Authors:** Moni Rani, Ratindra Nath Mondal, Rabiul Islam, Rakibul Islam, Annesha Saha Aishi, Arnab Saha, Md Abdur Razzak Sarker, Shah Md Sarwer jahan

**Affiliations:** 1 Radiotherapy, Rangpur Medical College, Rangpur, BGD; 2 Medicine, Prime Medical College, Rangpur, BGD; 3 Medical Strategy, Noora Health, Dhakka, BGD; 4 Medicine, Sir Salimullah Medical College, Mitford Hospital, Dhakka, BGD; 5 Communication and Media, Minnesota State University, Mankato, Mankota, USA; 6 Medicine, Dinajpur Medical College, Dinajpur, BGD

**Keywords:** bangladesh., breast cancer, cultural barriers, delayed diagnosis, treatment delay

## Abstract

Background

Breast cancer remains a major public health concern in low- and middle-income countries (LMIC's), including Bangladesh, where delayed healthcare-seeking contributes to late-stage diagnosis and poor outcomes. This study aimed to identify factors associated with delay in seeking medical care among breast cancer patients in Bangladesh.

Methods

A cross-sectional study was conducted at the Department of Radiotherapy, Rangpur Medical College Hospital, Bangladesh, from January to December 2024. A total of 206 newly diagnosed breast cancer patients aged ≥ 18 years were included. Data were collected using an interviewer-administered structured questionnaire covering socio-demographic characteristics, clinical features, presenting symptoms, healthcare-seeking behavior, and perceived causes of delay. Data were analyzed using IBM SPSS Statistics version 27. Descriptive statistics, Pearson’s chi-square test, and binary logistic regression were performed. Crude odds ratios (CORs) and adjusted odds ratios (AORs) with 95% confidence intervals were reported.

Results

The mean age of participants was 46 ± 11.0 years, and most patients were female (202, 98.0%). A breast lump was the presenting symptom in all patients. A large proportion of patients presented with advanced-stage disease, particularly Stage III (42.2%) and Stage IV (18.0%). Delay in seeking medical care was observed in 123 patients. The most common reported cause of delay was the use of traditional treatment/homeopathy (77.2%), followed by misinterpretation of symptoms as non-serious (24.4%). Cancer stage was significantly associated with delay in seeking medical care (p < 0.001). In the adjusted analysis, Stage IV disease was significantly associated with higher odds of delay [AOR = 10.8, 95% confidence interval (CI): 1.28-119, p = 0.039]. The strongest predictor of delay was first consultation with a homeopathy practitioner (AOR = 51.7, 95% CI: 19.7-163, p < 0.001). Socio-demographic factors, including age, residence, education, income, occupation, and smokeless tobacco use, were not significantly associated with delay.

Conclusion

Delay in seeking medical care among breast cancer patients in Bangladesh was mainly associated with initial consultation with homeopathy practitioners and advanced cancer stage, rather than socio-demographic factors. Strengthening public awareness, promoting early symptom recognition, improving referral pathways, and discouraging reliance on non-formal care pathways may help reduce delays in breast cancer diagnosis and treatment.

## Introduction

Breast cancer is the most prevalent cancer among women globally [1,2]. In 2020, approximately 16% of the 685,000 female deaths worldwide were attributed to breast cancer, highlighting its status as a significant health challenge [3]. While high-income countries have seen declining mortality rates, developing nations continue to experience elevated mortality rates due to limited screening facilities [2]. According to Bangabandhu Sheikh Mujib Medical University (BSMMU) [4], breast cancer accounts for 16.8% of cancer-related mortality in Bangladesh, indicating a substantial public health burden and the need for targeted interventions. Although the incidence of breast cancer is lower in low- and middle-income countries (LMICs) compared to high-income countries, mortality rates are disproportionately higher [3]. The incidence is increasing due to epidemiological transitions, with mortality projected to rise by 53.6% from 2020 to 2040 [3]. Factors such as limited healthcare access, lack of awareness, and sociocultural barriers contribute to delayed diagnoses and reduced survival rates [5,6].

In LMICs, delays in breast cancer care are categorized into two main types: patient delays and treatment delays. Patient delay refers to the period from the onset of symptoms to the seeking of medical care. This delay is influenced by various factors, including a lack of awareness, fear, cultural beliefs, and educational barriers [7,8]. Conversely, treatment delay is the time between diagnosis and the commencement of treatment, often resulting from systemic issues such as inadequate infrastructure and insufficient referrals [9]. Both types of delays significantly affect patient outcomes; notably, a four-week delay in initiating treatment is linked to a substantial increase in mortality risk [10]. Consequently, understanding the determinants of patient delay is critical for improving prognoses.

In Bangladesh, breast cancer is a leading cause of cancer-related deaths among women. According to Global Cancer Observatory (GLOBOCAN) [11], it accounts for 32.8% of all cancer-related fatalities and 69% of all female cancer cases, with similar trends anticipated for 2025. Nearly 90% of patients receive their diagnosis at advanced stages (III/IV), resulting in poor outcomes, higher treatment expenses, and lower survival rates. Detecting the disease early is crucial for enhancing prognosis and increasing survival chances [12,13]. Although research has examined awareness of breast self-examination [14] and obstacles to screening [15], evidence regarding factors associated with delays in seeking medical care and treatment remains limited, particularly within the Bangladeshi context [9,16,17]. This study sought to identify the socio-economic, cultural, and healthcare factors associated with delay in seeking medical care, operationally defined as seeking care more than three months after symptom onset among breast cancer patients in Bangladesh, aiming to inform policies for early detection and improved survival rates.

## Materials and methods

Study design, study setting, and participants

A hospital-based cross-sectional study was conducted at the Department of Radiotherapy, Rangpur Medical College Hospital, Bangladesh, from January 2024 to December 2024. A consecutive enrollment approach was used for participant recruitment. All newly diagnosed breast cancer patients attending the department during the study period were assessed for eligibility and invited to participate. For this study, “newly diagnosed” was operationally defined as having a diagnosis of breast cancer confirmed clinically and/or histopathologically within the two months preceding enrollment. Patients who fulfilled the inclusion criteria and provided informed consent were consecutively enrolled, resulting in a final sample of 206 participants. Patients were eligible if they were aged 18 years or older and had a new diagnosis of breast cancer confirmed either clinically or histopathologically. Participants were included regardless of their referral source from other healthcare facilities or previous history of benign breast disease. Patients were excluded if they had cognitive impairment, were unable to provide informed consent, were severely ill at the time of interview, had received a breast cancer diagnosis less than two months before enrollment, or were asymptomatic at the time of recruitment.

Ethical approval for this study was obtained from the Ethical Review Committee (ERC) of Rangpur Medical College, Bangladesh (Approval No. ERC-RPMC/2023/2339-C). Written informed consent was obtained from all participants before enrolment, and the study was conducted in accordance with the ethical principles of the Declaration of Helsinki.

Data collection and questionnaire design

The questionnaire was developed by a panel of experts based on prior literature [16,17] on breast cancer care and healthcare-seeking behavior, as well as their experience within Bangladesh's healthcare system (Appendix 1). The initial draft was revised through discussions among the co-investigators. A Bangla version (Appendix 2) was prepared using a forward-backward translation process performed by two independent translators to ensure conceptual consistency. The questionnaire was pre-tested among breast cancer patients who were not included in the final analysis, and necessary modifications were made to improve linguistic clarity and contextual relevance. Content validity was established through rigorous review by the expert panel. Pilot testing among 20 patients demonstrated good internal consistency (Cronbach’s alpha = 0.78) and excellent test-retest reliability (Cohen’s Kappa = 0.82).

Face-to-face interviews were conducted to collect data using a structured questionnaire administered by trained medical officers in the Department of Radiotherapy at Rangpur Medical College Hospital. Interviews were conducted with newly diagnosed breast cancer patients during their initial visit to the department. Participants were given sufficient time to respond, and interviewer influence was minimized to reduce information bias.

The questionnaire consisted of sections on sociodemographic information, clinical characteristics, symptom profile, and healthcare-seeking behavior. It collected information on age, sex, residence, marital status, educational level, occupation, monthly family income, smokeless tobacco use, comorbidities, presenting symptoms, first symptom noticed, time from symptom onset to first medical consultation, first healthcare provider consulted, reasons for delay in seeking care, delay in treatment initiation, reasons for treatment delay, and cancer stage at diagnosis.

The delay in seeking medical care was defined as the interval between the first recognition of breast-related symptoms-defined as the date the patient first noticed a physical change or symptom in the breast, such as a lump, pain, or skin changes-and the first consultation with a healthcare provider. A delay was considered present when this interval was more than three months. This > 3-month cutoff was justified based on established literature indicating that delays beyond 12 weeks are significantly associated with advanced-stage presentation and poorer survival outcomes in breast cancer [10,12]. Cancer staging was classified according to the tumor node metastasis (TNM) classification system, where Stages I and II are considered early-stage disease, and Stages III and IV are considered advanced-stage disease.

Data analysis

Data from 206 breast cancer patients were analyzed using IBM SPSS Statistics version 27.0 (IBM Corp., Armonk, USA). Descriptive statistics were employed to summarize socio-demographic, clinical, symptom-related, and healthcare-seeking characteristics. Categorical variables were expressed as frequencies and percentages, whilst continuous variables were presented as means ± standard deviations. The primary outcome variable was the delay in seeking medical care. Associations between categorical variables and delay were examined using Pearson’s chi-square test, with the χ² statistic, degrees of freedom, p-value, and Cramér’s V reported.

Variables for the multivariable logistic regression model were selected based on clinical relevance and a univariate p-value threshold of < 0.25. Before modeling, multicollinearity was assessed using the variance inflation factor (VIF), with all variables showing VIF < 2.0, indicating no significant multicollinearity. Model goodness-of-fit was evaluated using the Hosmer-Lemeshow test (p = 0.42), indicating a good fit. The sample size of 206 patients, with 123 events of delay, provided adequate statistical power (> 10 events per variable) for the variables included in the model. Binary logistic regression analysis was performed to estimate crude odds ratios and adjusted odds ratios with 95% confidence intervals for factors associated with delay in seeking medical care. No missing data were observed for the variables included in the statistical analyses.

## Results

Table 1 summarises the characteristics of 206 breast cancer patients, with a mean age of 46 ± 11 years. Most patients were middle-aged, particularly those aged 40-49 years (32%). The sample was predominantly female (98%) and had low educational attainment, with 57% having no formal education or ≤ 5 years of schooling. The majority were housewives (92%) and married (99%), and two-thirds (67%) belonged to a low-income group earning 5,001-10,000 Bangladeshi Taka (BDT). Smokeless tobacco use was reported in 36% of participants. Clinically, most patients presented at advanced stages, with Stage III the most common (42%), followed by Stage II (35%). Only 4.4% were diagnosed at Stage I and 18% at Stage IV, indicating late presentation.

**Table 1:** Socio-demographic and clinical characteristics of breast cancer patients (n = 206) Values are presented as frequency (n) and percentage (%). SD: standard deviation; BDT: Bangladeshi Taka; SLT: smokeless tobacco. Percentages may not sum to 100 due to rounding.

Variable	Frequency (n)	Percentage (%)
Age group (years)		
≤ 29	9	4.4
30–39	50	24.0
40–49	66	32.0
50–59	49	24.0
60–69	26	13.0
≥ 70	6	2.9
Mean ± SD	46 ± 11	—
Gender		
Male	4	1.9
Female	202	98.0
Education level		
Illiterate	59	29.0
≤ 5 years schooling	57	27.7
5–10 years schooling	59	28.6
10–12 years schooling	24	11.7
Graduate and above	7	3.4
Occupation		
Housewife	189	91.7
Service	10	4.9
Agriculture	7	3.4
Marital status		
Married	203	98.5
Unmarried	2	1.0
Monthly income (BDT)		
< 5000	10	4.9
5001–10000	137	66.5
10001–15000	38	18.4
> 15000	21	10.2
Smokeless tobacco use	74	35.9
Comorbidities		
None	162	78.6
Hypertension	16	7.8
Diabetes mellitus	11	5.3
Asthma	14	6.8
Other malignancy	3	1.5
Cancer stage		
Stage I	9	4.4
Stage II	73	35.4
Stage III	87	42.2
Stage IV	37	18.0

Table 2 shows symptom profiles and healthcare-seeking among 206 breast cancer patients. All (100%) had a breast lump; 98.1% noticed this first. Axillary lump (1.0%) and breast pain (1.0%) were rare initial symptoms. Most initially consulted homeopathy practitioners (46.1%), then surgeons (39.3%). Few saw gynaecologists (7.8%), medical specialists (2.4%), or went to GPs, government outpatient department (OPD), or pharmacies. Only 29.6% sought care within one month. Many delayed, with 30.1% taking three to six months, 16.0% six to 12 months, and 6.8% over 12 months, showing significant healthcare delay. 

**Table 2:** Symptom profile and healthcare-seeking behavior among breast cancer patients (n = 206) Values are presented as frequency (n) and percentage (%). SOB: shortness of breath; OPD: outpatient department. Percentages may not sum to 100 due to rounding.

Variable	Frequency (n)	Percentage (%)
Presenting symptoms		
Breast lump	206	100.0
Axillary lump	22	10.7
Metastatic symptoms (bone pain/SOB/headache/jaundice)	3	1.5
Nipple discharge	2	1.0
Other symptoms	4	1.9
First symptom noticed		
Breast lump	202	98.1
Breast pain	2	1.0
Axillary lump	2	1.0
First healthcare provider consulted		
Homeopathy practitioner	95	46.1
Surgeon	81	39.3
Gynecologist	16	7.8
Medicine specialist	5	2.4
General practitioner (GP)	2	1.0
Quack	2	1.0
Pharmacy/medicine shop	3	1.5
Government hospital OPD	1	0.5
Others	1	0.5
Time to seek medical care		
0–1 months	61	29.6
1–3 months	36	17.5
3–6 months	62	30.1
6–12 months	33	16.0
> 12 months	14	6.8

Table 3 summarises the main causes of delay in seeking care among breast cancer patients. The primary reason was traditional treatment (homeopathy) at 77.2%, followed by misinterpreting symptoms as non-serious at 24.4%. Other factors included financial constraints, belief symptoms would resolve spontaneously, stigma, and fear of diagnosis. For treatment delay, the main reason was “others” (32.1%), then financial constraints (25.0%) and fear of therapies (17.9%). A smaller portion reported delayed advice from doctors (14.3%), with no ongoing homeopathy use at this stage.

**Table 3:** Causes of delay in seeking medical care and treatment initiation (n = 206) Values are presented as frequency (n) and percentage (%). Note: The "Others" category for treatment delay included waiting for hospital bed availability, logistical/transport issues, and seeking second opinions.

Variable	Frequency (n)	Percentage (%)
Delay in seeking medical care (n = 123)		
Use of traditional treatment (homeopathy)	95	77.2
Misinterpretation of symptoms as non-serious	30	24.4
Financial constraints	5	4.1
Belief symptoms would resolve spontaneously	3	2.4
Social stigma	3	2.4
Fear of cancer diagnosis/treatment outcome	1	0.8
Others	2	1.6
Delay in treatment initiation (n = 28)		
Financial constraints	7	25.0
Fear of chemotherapy/radiotherapy/surgery	5	17.9
Doctor did not advise timely treatment	4	14.3
Continued use of homeopathy	0	0.0
Others	9	32.1

Table 4 shows that socio-demographic and clinical factors are linked to delays in seeking medical care among breast cancer patients. No significant association was found with residence (p = 0.946), education (p = 0.433), income (p = 0.943), occupation (p = 0.267), or smokeless tobacco use (p = 0.518), suggesting these do not influence delay. However, cancer stage was significantly associated (p < 0.001, V = 0.29); delayed care patients more often had advanced stages, especially Stage III (49%) and IV (23%). The strongest link was with the first healthcare provider (p < 0.001, V = 0.64); most delayed patients initially saw homeopathy practitioners (72%), while most non-delayed patients saw surgeons (72%), highlighting the initial care pathway as a key factor.

**Table 4:** Association between socio-demographic and clinical factors with delay in seeking medical care among breast cancer patients (n = 206) Statistical analysis used Pearson’s chi-square test; effect size was measured with Cramér’s V. Significance at p < 0.05.

Variable	No delay n = 83	Delay n = 123	χ²	DF	p-value	Effect size
Residence			0.00	1	0.946	0.00
Urban	19 (22.9%)	28 (23%)				
Rural	64 (77.1%)	95 (77%)				
Education level			0.61	1	0.433	0.05
High education	39 (47%)	51 (41%)				
Low education	44 (53%)	72 (59%)				
Monthly income (BDT)			0.01	1	0.943	0.00
High income	24 (29%)	35 (28%)				
Low income	59 (71%)	88 (72%)				
Occupation			1.23	1	0.267	0.08
Housewife	74 (89%)	115 (93%)				
Others	9 (11%)	8 (6.5%)				
Smokeless tobacco use (SLT)	32 (39%)	42 (34%)	0.42	1	0.518	0.05
Cancer stage			17.27	3	< 0.001	0.29
Stage I	6 (7.2%)	3 (2.4%)				
Stage II	41 (49%)	32 (26%)				
Stage III	27 (33%)	60 (49%)				
Stage IV	9 (11%)	28 (23%)				
First healthcare provider			83.35	2	< 0.001	0.64
Homeopathy	7 (8.4%)	88 (72%)				
Surgeon	60 (72%)	21 (17%)				
Others	16 (19%)	14 (11%)				

**Figure 1:**
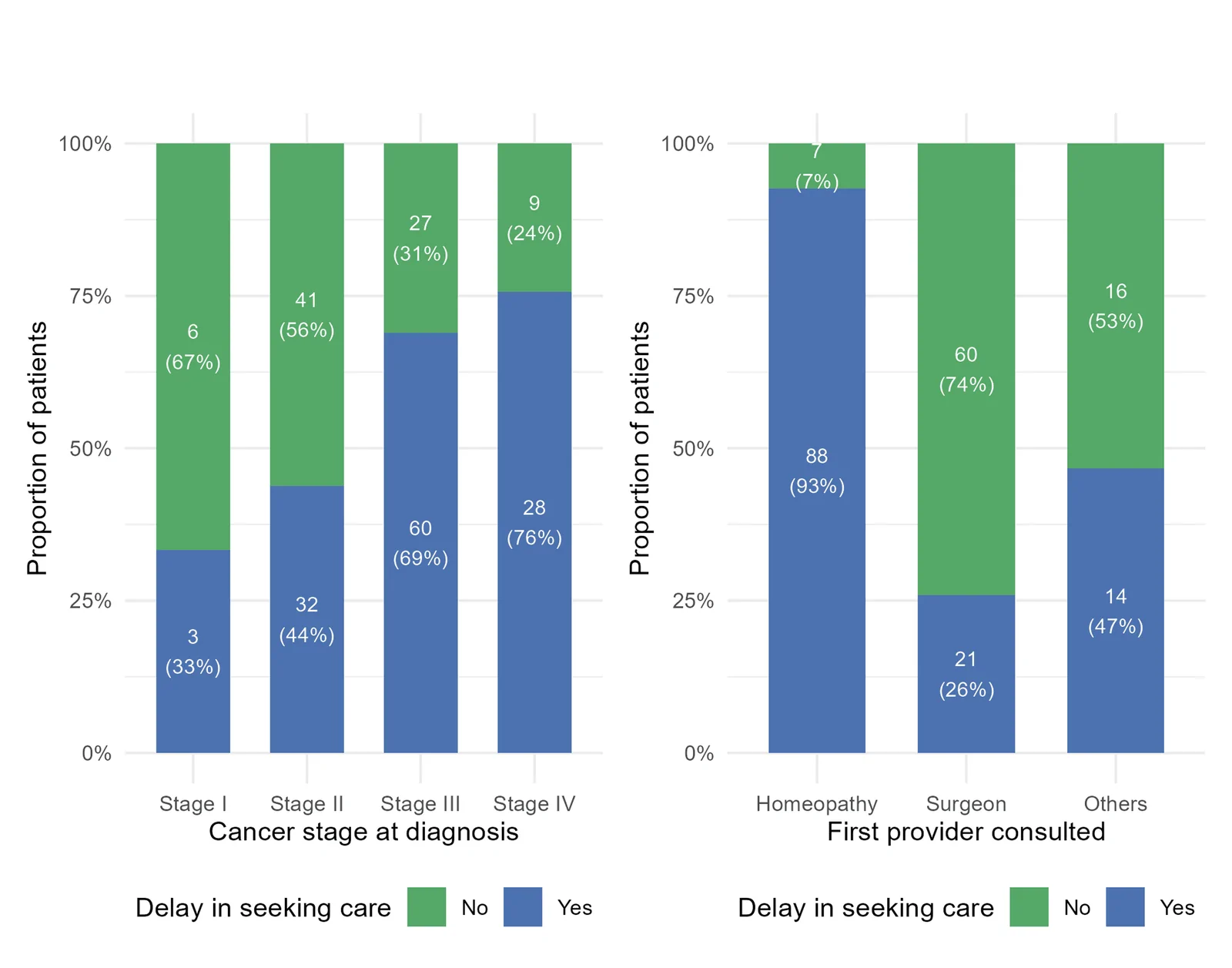
Care-seeking delay by cancer stage and first healthcare provider among breast cancer patients Figure 1 illustrates that delays in seeking medical care increase with advancing cancer stage, indicating a clear trend of late presentation in more advanced disease. It also shows that patients who first consult non-specialist providers, particularly alternative practitioners, are more likely to experience delayed care, whereas those who directly consult qualified surgeons tend to seek care earlier. While Tables 4 and 5 provide the statistical associations, Figure 1 visually synthesizes the intersection of cancer stage and provider type, offering an intuitive graphical representation of the compounded risk of delay.

Figure 1 illustrates that delays in seeking medical care increase with advancing cancer stage, indicating a clear trend of late presentation in more advanced disease. It also shows that patients who first consult non-specialist providers, particularly alternative practitioners, are more likely to experience delayed care, whereas those who directly consult qualified surgeons tend to seek care earlier.

Table 5 shows odds ratios for factors linked to delay in seeking care. After adjustment, Stage IV cancer significantly increased delay odds [adjusted odds ratio (AOR) = 10.8, p = 0.039], with Stage III borderline. The strongest predictor was first consultation with homeopathy practitioners (AOR = 51.7, p < 0.001), indicating a much higher delay risk. Other factors like age, residence, education, income, occupation, and smokeless tobacco use were not significantly linked to delay.

**Table 5:** Factors associated with delay in seeking medical care among breast cancer patients: crude and adjusted logistic regression analysis (n = 206) Crude odds ratios (COR) and adjusted odds ratios (AOR) with corresponding 95% confidence intervals (95% CI) derived from binary logistic regression analysis. Statistical significance was defined as p < 0.05. The reference categories used in the analysis were as follows: Stage I (cancer stage), urban residence (residence), high education (education level), high income (monthly income), housewife (occupation), no smokeless tobacco use, and other healthcare providers (first healthcare provider consulted).

	COR (95% CI)			AOR (95% CI)		
Variable	COR	95% CI	p-value	AOR	95% CI	p-value
Cancer stage						
Stage I	—	—		—	—	
Stage II	1.56	0.38, 7.85	0.550	2.33	0.31, 22.2	0.435
Stage III	4.44	1.09, 22.3	0.045	8.03	1.04, 81.9	0.060
Stage IV	6.22	1.36, 34.7	0.023	10.8	1.28, 119	0.039
Age						
≤ 29	—	—		—	—	
30–39	0.75	0.15, 3.20	0.706	1.12	0.15, 8.15	0.910
40–49	0.64	0.13, 2.64	0.549	1.10	0.15, 7.89	0.922
50–59	0.86	0.17, 3.70	0.845	1.13	0.14, 9.33	0.909
60–69	0.80	0.14, 3.80	0.784	0.60	0.06, 5.75	0.661
≥ 70	0.50	0.05, 4.21	0.521	0.23	0.01, 4.98	0.343
Residence						
Urban	—	—		—	—	
Rural	1.02	0.52, 1.98	0.946	1.08	0.40, 2.95	0.881
Education						
High education	—	—		—	—	
Low education	1.25	0.71, 2.20	0.433	1.76	0.66, 4.82	0.259
Monthly income						
High income	—	—		—	—	
Low income	1.02	0.55, 1.89	0.943	0.65	0.25, 1.63	0.360
Occupation						
Housewife	—	—		—	—	
Others	0.57	0.21, 1.56	0.272	0.48	0.10, 2.13	0.346
Smokeless tobacco use						
No	—	—		—	—	
Yes	0.83	0.46, 1.48	0.518	0.51	0.21, 1.19	0.126
First healthcare provider						
Surgeon	—	—		—	—	
Homeopathy	35.9	15.2, 96.9	< 0.001	51.7	19.7, 163	< 0.001
Others	2.50	1.04, 6.03	0.040	2.80	1.10, 7.27	0.032

## Discussion

Breast cancer remains a major public health challenge in LMICs, where delayed presentation continues to contribute to advanced-stage diagnosis, treatment complexity, and poorer survival outcomes. In Bangladesh, delays in breast cancer care are shaped not only by access barriers but also by patients’ early healthcare-seeking pathways. In the present study of 206 breast cancer patients, delayed seeking of medical care was observed in 123 patients, while 83 patients sought care without delay. The findings indicate that the initial choice of healthcare provider, particularly consultation with homeopathy practitioners, was the most important determinant of delay and was strongly associated with delay, whereas conventional socio-demographic factors showed limited influence.

A key finding of this study was the strong association between first healthcare provider consulted and delay in seeking medical care. Nearly half of patients initially consulted homeopathic practitioners, and among delayed patients, 72% first sought care from homeopathic providers, compared with only 8.4% among those without delay. In adjusted analysis, patients who initially consulted homeopathic practitioners had markedly higher odds of delayed care-seeking compared with those who consulted other providers [AOR = 51.7, 95% confidence interval (CI): 19.7-163, p < 0.001]. This suggests that the first point of contact after symptom recognition may be associated with whether patients enter an appropriate cancer care pathway or remain outside formal diagnostic services. Similar evidence from Malaysia has shown that complementary and alternative medicine use is associated with delayed presentation and diagnosis among breast cancer patients [18]. Earlier institutional findings also suggest that inadequate awareness and ineffective communication of breast cancer warning signs contribute to late presentation in LMIC settings [19]. Delayed consultation with qualified healthcare professionals has also been linked to late-stage diagnosis and poorer outcomes among breast cancer patients [20]. Together, these findings indicate that alternative treatment use should not be viewed only as a cultural preference, but as a major barrier that can delay diagnosis and worsen outcomes.

It is important to acknowledge that the exceptionally large adjusted odds ratio observed for homeopathy consultation (AOR = 51.7) reflects the strong polarization of care-seeking pathways in this setting, where patients who consult alternative practitioners often remain in these non-formal pathways for extended periods. While this magnitude seems unusually large, it is mathematically driven by the stark difference in delay proportions (72% vs. 8.4%). However, the very wide 95% confidence interval (15.7-130) indicates imprecision and possible sparse-data bias due to small subgroup sample sizes, and this finding should be interpreted with caution.

Breast lumps were the predominant clinical presentation, with 98.1% of participants identifying this as their primary warning sign. Despite this overt manifestation, a substantial proportion of the cohort failed to seek timely professional medical consultation. These delays were primarily driven by the use of traditional or homeopathic medicine, compounded by the underestimation of the symptom's gravity. This suggests that symptom identification is a necessary but insufficient condition for early diagnosis; effective outcomes depend upon the accurate appraisal of symptom significance and prompt engagement with formal healthcare systems. These findings corroborate existing literature indicating that diagnostic delays are often mediated by cultural paradigms, psychological responses, and the minimization of symptom severity [21]. Although stigma and apprehension regarding a cancer diagnosis were less prevalent in this cohort, psychological factors, specifically the fear of confirming malignancy and potential treatment outcomes, remain significant barriers to timely help-seeking behavior [22].

While financial constraints accounted for a smaller proportion of delays in initial care-seeking, they remained a critical factor in the initiation of treatment. Among patients experiencing delays in commencing treatment, 25.0% cited financial impediments, while 17.9% reported fear of chemotherapy, radiotherapy, or surgery as primary concerns. Within the Bangladeshi healthcare context, cancer management requires repeated clinical visits, diagnostic investigations, travel, and other ancillary expenses, placing substantial economic pressure on patients and their families. Evidence from LMIC's suggests that financial limitations restrict timely access to breast cancer care [23], and local research has similarly demonstrated the significant economic burden placed upon cancer survivors in Bangladesh [24]. Although income was not statistically associated with delay in the present study, financial vulnerability may nonetheless influence patient decision-making indirectly through deferred referrals, reliance on lower-cost alternative treatments, or the postponement of treatment initiation.

The current findings reveal that treatment initiation delays were less frequent than delays in initial healthcare seeking, indicating that the primary temporal gap occurs before patients access formal cancer care. Impediments to commencing treatment were largely attributed to financial burdens, trepidation concerning therapeutic modalities, and lapses in clinical guidance. Although data regarding treatment delay were collected, the small number of patients experiencing treatment delay (n = 28) precluded the performance of a robust multivariable regression analysis; therefore, these data were presented descriptively. Future multi-center studies with larger samples are recommended to explore treatment delay factors. These observations align with broader evidence suggesting that delayed therapy is driven by a complex interplay of patient-specific factors and healthcare system inefficiencies [25]. Collectively, these results highlight the critical need to bolster referral pathways and compress the timeframe between symptom recognition, formal diagnosis, and the initiation of appropriate treatment.

Clinical staging at presentation displayed a robust, statistically significant association with delays in care-seeking. Individuals experiencing delays were disproportionately prone to presenting with advanced-stage disease; specifically, 49% and 23% of the delayed cohort presented with Stage III and Stage IV disease, respectively, compared to 33% and 11% among their non-delayed counterparts. Multivariate adjusted analysis reinforced these findings, identifying Stage IV disease as a significant, independent predictor of delayed presentation relative to Stage I, while Stage III demonstrated a borderline effect. It should be noted that while cancer stage was included in the regression model to demonstrate this association, advanced stage is fundamentally a consequence of delay rather than a baseline predictor. The cross-sectional design limits causal inference, and reverse causation is an inherent limitation of this analysis. These findings align with established literature linking delayed medical engagement with advanced-stage breast cancer and elevated mortality risks [5,10]. Given that late-stage diagnoses necessitate more aggressive, complex, and costly therapeutic interventions, further exacerbating the financial impediments previously noted, there is an urgent clinical mandate to prioritize early detection and streamline care-seeking pathways [2].

A notable observation is the lack of statistically significant associations between delayed presentation and various socio-demographic variables. Neither geographic residence, educational attainment, monthly income, occupation, nor the use of smokeless tobacco demonstrated a significant correlation with delays; furthermore, these factors failed to emerge as independent predictors in the adjusted logistic regression analyses. This observation diverges from previous literature, which often cites lower education, economic instability, and rural residency as key determinants of treatment delay [6,11,16,24]. Conversely, the present data indicate that delays in this setting may be more strongly influenced by care-seeking pathways and symptom interpretation than by socio-demographic characteristics alone. Accordingly, interventions should not be confined to vulnerable or rural populations but should instead target community-wide health beliefs and care-seeking behaviors across all socio-demographic strata.

These observations underscore broader deficits regarding breast cancer literacy and early diagnostic engagement. Although breast masses were nearly universally identified as the primary symptom, a significant proportion of the cohort deferred seeking medical evaluation. This discrepancy implies that while symptom recognition is high, patients may misjudge the clinical gravity of the condition or opt for suboptimal initial care pathways. Such findings resonate with existing literature from Bangladesh, where insufficient health literacy and misconceptions significantly impede screening behaviors [26]. Broader research from Oman and other LMIC's similarly underscores psychosocial barriers, namely embarrassment, apprehension, and limited awareness, as critical impediments to timely help-seeking [27,28]. Consequently, there is an urgent imperative for culturally tailored public health interventions that emphasize the necessity of immediate clinical assessment for all breast irregularities, regardless of pain.

From a health systems perspective, the choice of initial provider carries significant policy implications. Given that individuals frequently consult informal or alternative practitioners such as homeopaths or medicine sellers prior to accessing formal health facilities, public health strategies must integrate these first-contact providers into early referral networks. Expediting the referral of patients presenting with breast masses to specialized medical facilities is imperative, as systemic inefficiencies and delays often exacerbate patient-level barriers, thereby worsening oncological outcomes [29,30]. Consequently, strengthening primary care recognition, streamlining referral protocols, and cultivating public trust in formal cancer services are critical strategies for reducing the incidence of late-stage presentation.

Several limitations merit consideration regarding this study. The cross-sectional design inherently precludes the establishment of causal relationships, while reliance on self-reported data introduces potential reporting biases. Specifically, because the interval between symptom onset and first consultation was based on patient recall, recall bias is an important limitation; participants may inaccurately remember the exact timing of symptom onset, potentially leading to the misclassification of delay status. Furthermore, as a single-center, hospital-based study including only patients who eventually reached a tertiary care facility, this study is subject to selection (referral) bias. Women who never accessed specialist care or died before referral were not represented, which may limit the generalizability of the findings. Additionally, several important confounding factors such as breast cancer awareness, family history, distance to healthcare facilities, screening history, health literacy, contraceptive drug use, and detailed clinical tumor characteristics (e.g., size, location, and number of lumps) were not evaluated. These should be acknowledged as potential sources of residual confounding. Finally, the absence of formal psychometric validation of the questionnaire in the broader population represents a methodological limitation.

## Conclusions

The current research identifies that delayed breast cancer care in Bangladesh is primarily associated with suboptimal healthcare-seeking pathways, specifically the initial consultation of informal providers rather than socio-demographic variables, driven by suboptimal healthcare-seeking pathways, specifically the initial consultation of informal providers rather than socio-demographic variables, with such delays demonstrating a significant correlation with advanced disease presentation. These findings underscore the critical imperative to enhance public awareness regarding symptom recognition, optimize referral protocols, and integrate non-formal first-contact practitioners into established oncological care networks to mitigate delays and improve clinical prognosis.

## Appendices

Appendix 1 

**Table 6:** English questionnaire used in the study Before starting the interview, the researcher stated that they would like to begin by asking you some background questions, followed by questions about your breast-related symptoms, diagnosis, and healthcare-seeking experience. All information you provide will be kept confidential and used only for research purposes.

Question No.	Question	Possible Response With Instructions
1	Age	Record completed age in years: ______ years. Later categorize as: ≤ 29 = 1, 30–39 = 2, 40–49 = 3, 50–59 = 4, 60–69 = 5, ≥ 70 = 6
2	Gender	Male = 1, Female = 2
3	Residence	Urban = 1, Rural = 2
4	Marital status	Married = 1, Unmarried = 2
5	Education level	Illiterate = 1, ≤ 5 years schooling = 2, 5–10 years schooling = 3, 10–12 years schooling = 4, Graduate and above = 5. For analysis: Low education = illiterate/ ≤ 5 years; High education = > 5 years
6	Occupation	Housewife = 1, Service = 2, Agriculture = 3, Others = 4
7	Monthly family income	< 5000 BDT = 1, 5001–10000 BDT = 2, 10001–15000 BDT = 3, > 15000 BDT = 4. For analysis: Low income = ≤ 10000 BDT; High income = > 10000 BDT
8	Smokeless tobacco use	Yes = 1, No = 2
9	Comorbidities	None = 0, Hypertension = 1, Diabetes mellitus = 2, Asthma = 3, Other malignancy = 4. If more than one is present, record all applicable responses
10	Presenting symptoms	Breast lump = 1, Axillary lump = 2, Metastatic symptoms = 3, Nipple discharge = 4, Other symptoms = 5. Multiple responses may be selected
11	First symptom noticed	Breast lump = 1, Breast pain = 2, Axillary lump = 3
12	Time from first symptom onset to first medical care	0–1 month = 1, 1–3 months = 2, 3–6 months = 3, 6–12 months = 4, > 12 months = 5
13	Delay in seeking medical care	No delay = 0, if medical care was sought within ≤ 3 months of symptom onset. Delay = 1, if medical care was sought after > 3 months of symptom onset
14	First healthcare provider consulted	Homeopathy practitioner = 1, Surgeon = 2, Gynecologist = 3, Medicine specialist = 4, General practitioner = 5, Quack = 6, Pharmacy/medicine shop = 7, Government hospital OPD = 8, Others = 9. For analysis: Homeopathy = 1, Surgeon = 2, Others = 3
15	Causes of delay in seeking medical care	Use of traditional treatment/homeopathy = 1, Misinterpretation of symptoms as non-serious = 2, Financial constraints = 3, Belief symptoms would resolve spontaneously = 4, Social stigma = 5, Fear of cancer diagnosis/treatment outcome = 6, Others = 7. Select all applicable responses
16	Time from confirmed diagnosis to start of treatment	Record duration: ______ days/weeks/months
17	Delay in treatment initiation	No delay = 0, if treatment was started within ≤ 3 months after diagnosis. Delay = 1, if treatment was started after > 3 months after diagnosis
18	Causes of delayed treatment initiation	Financial constraints = 1, Fear of chemotherapy/radiotherapy/surgery = 2, Doctor did not advise timely treatment = 3, Continued use of homeopathy = 4, Others = 5. Select all applicable responses
19	Cancer stage at diagnosis	Stage I = 1, Stage II = 2, Stage III = 3, Stage IV = 4

Appendix 2 

**Table 7:** Bangla version of the questionnaire used in the study আপনার স্বাস্থ্য সম্পর্কিত প্রশ্ন করার আগে আমি কিছু প্রাথমিক তথ্য জানতে চাই। এই তথ্য সম্পূর্ণ গোপনীয় রাখা হবে এবং শুধুমাত্র গবেষণার উদ্দেশ্যে ব্যবহার করা হবে।

প্রশ্ন নং	প্রশ্ন	সম্ভাব্য উত্তর ও নির্দেশনা
১	বয়স	সম্পূর্ণ বয়স বছর হিসেবে লিখুন: ______ বছর। পরে শ্রেণিবিভাগ করুন: ≤ ২৯ = ১, ৩০–৩৯ = ২, ৪০–৪৯ = ৩, ৫০–৫৯ = ৪, ৬০–৬৯ = ৫, ≥ ৭০ = ৬
২	লিঙ্গ	পুরুষ = ১, মহিলা = ২
৩	বসবাসের স্থান	শহর = ১, গ্রাম = ২
৪	বৈবাহিক অবস্থা	বিবাহিত = ১, অবিবাহিত = ২
৫	শিক্ষাগত যোগ্যতা	নিরক্ষর = ১, ≤ ৫ বছর স্কুলিং = ২, ৫–১০ বছর স্কুলিং = ৩, ১০–১২ বছর স্কুলিং = ৪, স্নাতক বা তার বেশি = ৫। বিশ্লেষণের জন্য: কম শিক্ষা = নিরক্ষর/≤ ৫ বছর; উচ্চ শিক্ষা = > ৫ বছর
৬	পেশা	গৃহিণী = ১, চাকরি = ২, কৃষি = ৩, অন্যান্য = ৪
৭	মাসিক পারিবারিক আয়	< ৫০০০ টাকা = ১, ৫০০১–১০০০০ টাকা = ২, ১০০০১–১৫০০০ টাকা = ৩, > ১৫০০০ টাকা = ৪। বিশ্লেষণের জন্য: কম আয় = ≤ ১০০০০ টাকা; উচ্চ আয় = > ১০০০০ টাকা
৮	ধোঁয়াবিহীন তামাক ব্যবহার	হ্যাঁ = ১, না = ২
৯	সহ-রোগ	নেই = ০, উচ্চ রক্তচাপ = ১, ডায়াবেটিস মেলিটাস = ২, হাঁপানি = ৩, অন্য কোনো ক্যান্সার = ৪। একাধিক রোগ থাকলে প্রযোজ্য সবগুলো উত্তর লিখুন
১০	উপস্থিত উপসর্গ	স্তনে চাকা/লাম্প = ১, বগলে চাকা/লাম্প = ২, মেটাস্ট্যাটিক উপসর্গ = ৩, নিপল ডিসচার্জ = ৪, অন্যান্য উপসর্গ = ৫। একাধিক উত্তর নির্বাচন করা যাবে
১১	প্রথম যে উপসর্গটি লক্ষ্য করেছিলেন	স্তনে চাকা/লাম্প = ১, স্তনে ব্যথা = ২, বগলে চাকা/লাম্প = ৩
১২	প্রথম উপসর্গ দেখা দেওয়া থেকে প্রথম চিকিৎসা নেওয়া পর্যন্ত সময়	০–১ মাস = ১, ১–৩ মাস = ২, ৩–৬ মাস = ৩, ৬–১২ মাস = ৪, > ১২ মাস = ৫
১৩	চিকিৎসা নিতে বিলম্ব	বিলম্ব নেই = ০, যদি উপসর্গ দেখা দেওয়ার ≤ ৩ মাসের মধ্যে চিকিৎসা নেওয়া হয়। বিলম্ব আছে = ১, যদি উপসর্গ দেখা দেওয়ার > ৩ মাস পরে চিকিৎসা নেওয়া হয়
১৪	প্রথম যে স্বাস্থ্যসেবা প্রদানকারীর কাছে গিয়েছিলেন	হোমিওপ্যাথি চিকিৎসক = ১, সার্জন = ২, গাইনোকোলজিস্ট = ৩, মেডিসিন বিশেষজ্ঞ = ৪, জেনারেল প্র্যাকটিশনার = ৫, কোয়াক/হাতুড়ে চিকিৎসক = ৬, ফার্মেসি/ওষুধের দোকান = ৭, সরকারি হাসপাতালের বহির্বিভাগ = ৮, অন্যান্য = ৯। বিশ্লেষণের জন্য: হোমিওপ্যাথি = ১, সার্জন = ২, অন্যান্য = ৩
১৫	চিকিৎসা নিতে বিলম্বের কারণ	প্রচলিত/হোমিওপ্যাথি চিকিৎসা গ্রহণ = ১, উপসর্গকে গুরুতর মনে না করা = ২, আর্থিক সমস্যা = ৩, উপসর্গ নিজে নিজে ভালো হয়ে যাবে বলে মনে করা = ৪, সামাজিক কলঙ্ক/লজ্জা = ৫, ক্যান্সার নির্ণয় বা চিকিৎসার ফলাফল নিয়ে ভয় = ৬, অন্যান্য = ৭। প্রযোজ্য সবগুলো উত্তর নির্বাচন করুন
১৬	নিশ্চিত রোগনির্ণয় থেকে চিকিৎসা শুরু পর্যন্ত সময়	সময় লিখুন: ______ দিন/সপ্তাহ/মাস
১৭	চিকিৎসা শুরু করতে বিলম্ব	বিলম্ব নেই = ০, যদি রোগনির্ণয়ের ≤ ৩ মাসের মধ্যে চিকিৎসা শুরু হয়। বিলম্ব আছে = ১, যদি রোগনির্ণয়ের > ৩ মাস পরে চিকিৎসা শুরু হয়
১৮	চিকিৎসা শুরু করতে বিলম্বের কারণ	আর্থিক সমস্যা = ১, কেমোথেরাপি/রেডিওথেরাপি/সার্জারি নিয়ে ভয় = ২, চিকিৎসক সময়মতো চিকিৎসার পরামর্শ দেননি = ৩, হোমিওপ্যাথি চিকিৎসা চালিয়ে যাওয়া = ৪, অন্যান্য = ৫। প্রযোজ্য সবগুলো উত্তর নির্বাচন করুন
১৯	রোগনির্ণয়ের সময় ক্যান্সারের স্টেজ	স্টেজ I = ১, স্টেজ II = ২, স্টেজ III = ৩, স্টেজ IV = ৪
